# Advances in Human Immune System Mouse Models for Studying Human Hematopoiesis and Cancer Immunotherapy

**DOI:** 10.3389/fimmu.2020.619236

**Published:** 2021-02-02

**Authors:** Syed A. Mian, Fernando Anjos-Afonso, Dominique Bonnet

**Affiliations:** ^1^Haematopoietic Stem Cell Lab, The Francis Crick Institute, London, United Kingdom; ^2^Department of Haematology, School of Cancer and Pharmaceutical Sciences, King’s College London, London, United Kingdom; ^3^Haematopoietic Signalling Group, European Cancer Stem Cell Institute, School of Biosciences, Cardiff University, Cardiff, United Kingdom

**Keywords:** immunodeficient mice models, immunotherapy, human hematopoiesis, xenotransplantation models, immune reconstitution

## Abstract

Immunotherapy has established itself as a promising tool for cancer treatment. There are many challenges that remain including lack of targets and some patients across various cancers who have not shown robust clinical response. One of the major problems that have hindered the progress in the field is the dearth of appropriate mouse models that can reliably recapitulate the complexity of human immune-microenvironment as well as the malignancy itself. Immunodeficient mice reconstituted with human immune cells offer a unique opportunity to comprehensively evaluate immunotherapeutic strategies. These immunosuppressed and genetically modified mice, with some overexpressing human growth factors, have improved human hematopoietic engraftment as well as created more functional immune cell development in primary and secondary lymphoid tissues in these mice. In addition, several new approaches to modify or to add human niche elements to further humanize these immunodeficient mice have allowed a more precise characterization of human hematopoiesis. These important refinements have opened the possibility to evaluate not only human immune responses to different tumor cells but also to investigate how malignant cells interact with their niche and most importantly to test immunotherapies in a more preclinically relevant setting, which can ultimately lead to better success of these drugs in clinical trials.

## Brief History of Human Hematopoiesis

HSCs are rare (one in 10^6^ bone marrow cells) undifferentiated multipotent stem cells with the ability to perpetuate themselves for indefinite time through self-renewal. These HSCs differentiate to all the downstream mature blood cell lineages that control the homeostasis balance, immune function, and response to infections. Since the pioneering work of Till and McCulloch in the early 1960s, the understanding of HSCs has progressed enormously from observational to functional studies. The regenerative potential of HSCs was further established with the development of various clonal repopulation assays pioneered by Becker et al. (1), McCulloch et al. (2, 3), Moore and Metcalf (4, 5), Griffin and Löwenberg (6) and Dicke et al. (7). Even though our understanding of hematopoiesis largely comes from the mouse system, our ability to characterize human HSCs has improved. Since the discovery of a ligand for L-selectin (namely CD34) by Civin et al. (8), that for decades became the cornerstone, cell surface marker for enriching human HSCs, is still being used alongside newly well-established antigens (such as CD38, CD45RA, CD90, and CD49f) (9–15). However, the concept of SCID-repopulating cell (SRC) assay by Dick and colleagues was the game changing idea that enabled to accurately measure the primitive HSC populations using NOD/SCID mice (16, 17). Altogether, thanks to the use of robust *in vivo* and *in vitro* clonal assays, xenotransplantation as well as refined sorting strategies using cell surface antigens, significant insights toward understanding the human hematopoietic hierarchy have been made. Today it is well established that human HSCs are present within the CD34^+^CD38^−^CD90^+^CD45RA^−^CD49f^+^ fraction of the hematopoietic compartment, with an SRC frequency of one in 10 cells (15). There is also emerging evidence suggesting the existence of some HSCs within the CD34^-^ faction of the human bone marrow cells, however the frequency of these HSCs is low (18–21).

## A Brief Note on the Development and Refinement of Immunodeficient Mouse Models to Study Human Hematopoiesis

Gaining knowledge of human physiology and pathophysiology has been often hampered by restricted access to human tissues or limited to performing *in vitro* assays. Over the last few decades, advances made from inbred wild-type to more state-of-the-art genetically engineered humanized strains have enabled researchers to gain novel insights into the complex biological underpinnings of human hematopoiesis. The development of humanized mouse models started with the identification of the severe combined immunodeficient (Scid; mutation in *Prkdc^scid^*) mice that lacked B and T cells (22). These mice were shown to allow human T and B cell reconstitution (23). However, the SCID mouse model has limited usage due to the spontaneous generation of mouse T and B cells during aging (also known as leakiness) and high levels of host NK cell activity, that limits the engraftment of human cells. Further attempts to modify the SCID mouse model were made to increase the human hematopoietic cell engraftment which eventually led to the development of the non-obese diabetic-SCID (NOD-SCID) mice. This hybrid model was developed by backcrossing the SCID mice onto the non-obese diabetic (NOD) mouse background, which resulted in immunological multi-dysfunction, including defective NK cell activity and complement-dependent hemolytic activity (24). The NOD background is supportive of human cells due to a specific mutation in *Sirpa* that confers high affinity to human CD47, resulting in host macrophage tolerance to human cells (25). These NOD-*Scid* mice supported high levels of human cell engraftment compared to other non-NOD derived immunodeficient mice. However, the use of this hybrid NOD-SCID mice remained limited due to relatively short life span (median survival = 257 days) as well as the residual activity of NK cell and some of the other innate components (26, 27) of the immune system, thereby impeding the engraftment of the human cells. These mice also have a propensity to developing thymic lymphomas with age, similar to SCID mice.

**Figure 1:**
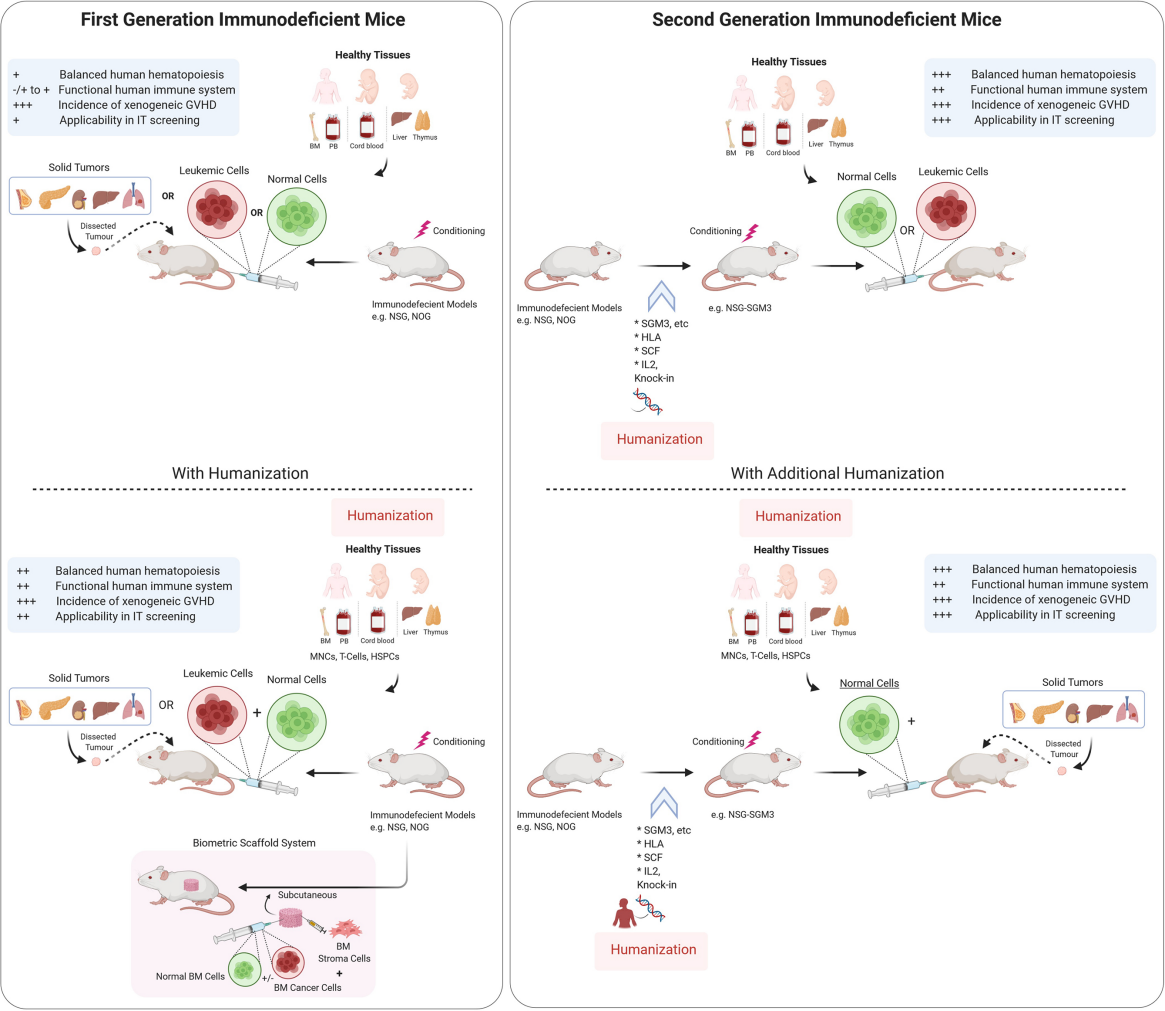
Schematic representation of Immunodeficient mouse models and humanization strategies used to establish preclinical models. Tumor tissues from cancer patients are either injected (for hematological cancers) or implanted subcutaneously or orthotopically into immunodeficient mice with or without humanization. Scaffold implantation approach has also be used for hematological cancers in mice. Immunodeficient mouse models are either used for understanding the normal hematopoiesis or pathophysiology of the cancer tissues and for testing theurapeutic strategies. There are varying degrees of benefits for using different immunodeficient mouse models as depecited in the panel (+/−). IT, Immunotherapy, GVHD, Graft *vs* host disease, I.V., Intravenous route, BM, bone marrow, HSPC, hematopoietic stem and progenitor cells. Illustration was created with Biorender.com.

The next revolution in the development of advanced immunodeficient mice occurred with the backcrossing of NOD-*Scid* mice with either truncated (NOG) or deleted (NSG) interleukin-2 receptor (IL-2R) common *γ* chain. Both of these models have deficiency in the IL-2R *γ*-chain that leads to the multiple defects in the innate as well as the adaptive immune system and prevents NK cell development (28–30). In addition, the lack of the IL-2R*γ*c activity limits the spontaneous development of thymoma, therefore allowing for long-term human cell engraftment experiments (median survival >89 weeks) (31). Although NOD-*Scid*, NOG, and NSG mice are the three most commonly used models for xenotransplantation, there are also other strains that have been developed over the years. For example, Rag-deficient mice that lack the expression of functional Rag1 or Rag2 proteins cannot perform somatic recombination of the T cell receptor (TCR) and immunoglobulin (Ig) genes, and the absence of either of these genes results in T and B cell deficiency. Mice that carry either the *Rag1* or *Rag2* mutations have very similar phenotypes and are also commonly used nowadays (BALB/c-*Rag2*^−/−^*Il2rγ*^−/−^ or BRG and the NOD-*Rag1*^−/−^*Il2rγ*^−/−^ or NRG mice). Despite these improvements, all of the hybrid Il2r*γ*^–/–^ mice strains still require conditioning with either sublethal radiation or with a myeloablative agent such as bulsulfan prior to cell injection of low number of human cells for optimal engraftment. Further detailed reviews on the historical development of the different mouse models have been extensively covered elsewhere (32–34). Instead, we will critically discuss the recent advancements and pitfalls concerning the development of the different human blood cells in these mice, particularly focusing on their use to investigate cancer immunotherapies (**Figure 1**). Nevertheless, a brief description of such models described here is summarized in **Table 1**.

**Table 1:** Summary of the immunodeficient mouse strains developed over the last few decades described in this review.

Name of the strain (genetic description) (reference when first described)	Features of the mice
***BRG*** or **BALB/c-*Rag2*^−/−^***Il2r* **γ^−/−^** *(*C.Cg-*Rag2^tm1Fwa^ Il2rg^tm1Sug^*/JicTac (Taconic) or **C;129S4-*Rag2^tm1.1Flv^ Il2rg^tm1.1Flv^*/J** (Jackson)) (35)	*-*BALB/c background with *Rag2 and Il2rg* knockout; Taconic strain is completely congenic on BALB/c background whereas Jackson is on a 129 background -both models have higher radiation tolerance due to *Rag2* mutation compared to Scid models -both models lack T, B, and NK cells and dysfunctional DCs
**BRG-*IL3*/*CSF2*** (C;129S4-*Rag2^tm1.1Flv^ Csf2*/*Il3^tm1.1(CSF2,IL3)Flv^ Il2rg^tm1.1Flv^*/J) (36)	*-*similar to BRG *-*targeted replacement of mouse *Il3* and *Csf2* with coding regions with those of human *-*improve human myeloid cell reconstitution in the lung in particular alveolar macrophages
**BRGS** (C;129S4-Rag2^tm1.1Flv^ IL2rg^tm1.1F1v^Tg(SIRPA)1Flv (37)	*-*similar to BRG *-*heterozygous human *SIRPA* BAC transgene expression -allow better engraftment and maintenance of human hematopoietic cells compared to BRG mice to levels comparable to NSG mice
**BRG-*TPO* or *Rag2*^−/−^*Il2ry*^−/−^-*TPO*** (C;129S4-*Rag2^tm1.1Flv^ Thpotm1.1(TPO)Flv Il2rgtm1.1Flv*)/J (38)	*-*similar to BRG *-*human *TPO* knock-in replacing mouse *Tpo* *-*support higher human myeloid-lymphoid and HSC engraftment compared to BRG -have reduced mouse platelets compared to BRG
***Rag2^−/−Il2ry−/−-^*SIRPA^h/m^*IL6^h/h^*** *or* **RGSKI-*IL6*** (39)	-similar to BRG -heterozygous human *SIRPA BAC* transgene expression and homozygous *IL6* knock-in replacing mouse *Il6* -CD47 expressed on human cells binds efficiently to the mouse BAC transgene-encoded human SIRPα enabling mouse phagocytes to tolerate and not engulf engrafted human cells -allow improved thymopoiesis and peripheral T cell reconstitution *-*allow higher frequencies of human memory and IgG producing B cells development with increased in total IgG and antigen-specific IgG levels
***Rag2^−/−Il2ry−/−-^*SIRPA^h/m^*IL15****^h/m^ or **SRG-15** (*40*)*	*-*similar to BRG *-*heterozygous human *SIRPA* knock-in -homozygous knock-in *IL15* replacing mouse *Il15* *-*expression of human SIRP*α* enables mouse phagocytes to tolerate and not engulf engrafted human cells *-s*upport efficient development of human circulating and tissue-resident NK cells, intraepithelial lymphocytes and innate lymphoid cell subsets
**MITRG** (C;129S4-*Rag2^tm1.1Flv^ Csf1^tm1(CSF1)Flv^ Csf2*/*Il3^tm1.1(CSF2,IL3)Flv^ Thpo^tm1.1(TPO)Flv^ Il2rg^tm1.1Flv^*/J) (41)	*-*similar to BRG *-human CSF1, CSF2/IL3* and TPO knock-in replacing the respective mouse genes -enable the full recapitulation of human myeloid development and function due to the expression of human cytokines
**MISTRG** (C;A29S4-*Rag2^tm1.1Flv^ Csf1^tm1^(CSF1)FlvCsf2^/Il3tm1.1^(CSF2,IL3)Flv Thpo^tm1.1^(TPO)Flv Il2rg^tm1.1Flv^* Tg(SIRPA)1Flv/J) (41)	-similar to MITRG -human *SIRPA* BAC transgene expression -CD47 expressed on human cells binds efficiently to the mouse BAC transgene-encoded human SIRPα enabling mouse phagocytes to tolerate and not engulf engrafted human cells
**MISTRG6** (42)	-similar to MISTRG -homozygous *IL6* knock-in replacing mouse Il6 -allow the development of entire spectrum of human plasma cell neoplasia
***Scid*** *(Prkdcscid) (*22*)*	*-*lack functional lymphocytes because of impaired VDJ rearrangement -have severe combined immunodeficiency affecting both B and T lymphocytes
**NOD-*****Scid*** *(*NOD.Cg-*Prkdc^scid^*/J) (24)	-as *Scid* mice *-*NOD/ShiJic strain background contributes to defective mouse DCs and macrophages -have reduced complement and NK cells -harbour *Sirpa* polymorphism that allows interactions between mouse macrophages and human CD47
**NOD-*****Scid*** **-SGM3** (NOD.Cg-*Prkdc^scid^* Tg(CMV-IL3,CSF2,KITLG)1Eav (43)	-as NOD-*Scid* -transgenic expression of human IL-3, GM-CSF, and SCF under the CMV promoter -support higher level of human myeloid cell engraftment -induce exhaustion of human HSCs
**NOG** (NOD.Cg-*Prkdc^scid^Il2rgtm^1Sug^*/JicTac) (29)	-features similar to NOD-*Scid* *-*truncated intracytoplasmic domain of IL-2Rg leading to no signalling -have defective mouse NK cell development
**NOG-EXL** or **NOG-*IL3*/*CSF2*** (NOD.Cg-*Prkdc^scid^Il2rg^tm1Sug^*(SV40/HTLV-IL3,CSF2)10-7Jic/JicTac) (44)	-similar to NOG -transgenic expression of human GM-CSF and IL-3 driven by the SRα promoter *-*support higher levels of human myeloid cell differentiation -support higher human DC and mast cells differentiation -allow more efficient human HSC engraftment
**NOG-*IL6*** (NOD.Cg-*Prkdc^scid^Il2rg^tm1Sug^*^Tg^(CMV-IL6)1-1Jic/JicTac) (45)	*-*similar to NOG -transgenic expression of human IL-6 driven by the CMV promoter -allow enhanced human monocytes development
**NOG-*IL15*** (NOD.Cg-*Prkdc^scid^Il2rg^tm1Sug^*^Tg^(CMV-IL2/IL15)1-1Jic/JicTac) (46)	*-*similar to NOG -transgenic expression of human IL-15 with a IL-2 signal peptide driven by the CMV promoter -allow extensive human NK cell proliferation and differentiation and human NK cells produce both granzyme A and perforin upon stimulation -allow engraftment and expansion of human NK cells following engraftment with CD56+ NK cells derived from human PBMCs
**NRG** or **NOD-*Rag1*^−/−^***Il2ry* **^−/−^** (NOD.Cg-*Rag1t^m1MomIl^2rg^tm1Wjl^*/*SzJ) (*47*)*	-harbor *Rag1^null^* and *IL2r*γ*^null^* mutations -lack T, B, and NK cells and dysfunctional DCs and macrophages -have *Sirpa* polymorphism that allows interactions between mouse macrophages and human CD47
**NRG-HLA-A2-DR4** or **DRAG** (NOD.Cg-*Rag1^tm1Mom^ Il2rg^tm1Wjl^* Tg(HLA-DRA,HLA-DRB1*0401)39-2Kito/ScasJ) (48)	-similar to NRG -express chimeric human-mouse class II transgenes HLA-DRA/HLA-DRB1*0401 fused to the *I-E^d^* MHC class II molecule -allow enhanced HLA-DR-matched HSC engraftment and subsequent human T cell and B cell development to study the development of autoimmune diseases -allow vaccine testing and generation of human IgM, IgG, IgA or IgE monoclonal antibodies
**NSG** (NOD.Cg-*Prkdc^scidIl^2rg^tm1Wjl^/*SzJ) (28)	-features similar *to* NOD-*Scid* *-*target mutation of Il2rg leading to no expression of IL-2 -have defective NK cell development
**NSG-*B2M^null^*** *(*NOD.Cg-*B2m^tm1Unc^ Prkdc^scid^ Il2rg^tm1Wjl^*/SzJ) (49)	*-*similar to NSG -are relatively resistant to GVHD
**NSG-*CSF1*** (NOD.Cg-*Prkdc^scid^ Il2rg^tm1Wjl^* Tg(CSF1)3Sz/SzJ) (50)	*-*similar to NSG -transgenic expression of human M-CSF *-*support higher human macrophage development
**NSG-*HLA-A2/HHD*** (NOD.Cg-*Prkdc^scid^ Il2rg^tm1Wjl^* Tg(HLA-A/H2-D/B2M)1Dvs/SzJ) (51)	*-*similar to NSG *-*express human HLA class 1 heavy and light chains with B2M covalently linked to the MHC class 1, alpha1 and alpha2 binding domains of the human *HLA-A2.1* gene, and the alpha3, cytoplasmic and transmembrane domains of the murine *H2-D^b^* -support T cell maturation and responses to human antigens presented by HLA-A2.1 molecule
**NSG-*HLA-A24/HHD*** (NOD.Cg-*Prkdc^scid^* Il2rg*^tm1Wjl^* Tg(HLA-A24/H2-D/B2M)3Dvs/Sz) (52)	*-*similar to NSG *-*express human *HLA-A24.2* with *B2M* and murine *H2-D^b^* -support human T cell maturation and responses to human antigens presented by HLA-A24.2 molecule
**NSG‐*HLA‐A2/DR1*** (NOD.Cg-*Prkdc^scid^ IL2rg^tmlWjl^/Sz* Tg[HLA-DRA*0101,HLA-DRB1*0101]1Dmz/GckRolyJ *Mcph1^Tg(HLA-A2.1)1Enge^*/SzJ (48)	*-*similar to NSG -express human/mouse chimeric MHC Class II transgene (Tg(HLA-DRB1*01)1Dmz): parts of mouse MHC Class II *H2-Ea* and *H2-Eb1* were replaced by the corresponding amino acids of the human MHC Class II protein -Tg(HLA-A2.1)1Enge integrated into chromosome 8 causing a duplication in *Mcph1* resulting into functional knock-out of *Mcph1* in homozygous mice -support human T cell maturation and responses to human antigens presented by the respective HLA molecules. *-*allow the engraftment of functional human Th1, Th2 and Th17 T cells
**NSG***-* **(*K^b^D^b^*)*null***(***IA^null^*)** (NOD.Cg-*Prkdc^scid^ H2-K1^tm1Bpe^ H2-Ab1^em1Mvw^ H2-D1^tm1Bpe^ Il2rg^tm1Wjl^*/SzJ) (49)	*-*similar to NSG -have MHC class I molecule (H2-K and D) and MHC class II deficiencies (IA) -are resistant to GVHD
**NSG-*IL6*** NOD.Cg-*Prkdc^scid^Il2rg^tm1Wjl^ Tg(IL6)/Sz)* (53)	*-*similar to NSG -transgenic expression of human IL-6 -allow higher levels of human CD3^+^ and Th17 T cell development -allow higher plasma levels of human IgM and IgG
**NSG-SGM3** (NOD.Cg-*Prkdc^scid^Il2rg^tm1Wjl^* Tg(CMV-IL3,CSF2,KITLG)1Eav/MloySzJ) (54)	*-*similar to NSG *-*transgenic expression of human IL-3, GM-CSF and SCF under the CMV promoter -induce exhaustion of human HSCs *-*allow higher level of human myeloid cell and AML engraftment -allow rapid reconstitution of human T cells, with improved B cell differentiation and increased levels of NK cells
**NSG-SGM3-*CSF1*** or **QUAD** (NOD.Cg-*Prkdc^scid^ Il2rg^tm1Wjl^* Tg(CMV-IL3,CSF2,KITLG)1Eav Tg(CSF1)3Sz/J) (33)	*-* similar to NSG-SGM3 combined with features of NSG-*CSF1*
**NSG Kit^W41/W41^** or **NSGW41** (NOD.Cg-*Kit^W-41J^ Prkdc^scid^ Il2rg^tm1Wjl^*/WaskJ) (55)	-similar to NSG *-*loss of endogenous *Kit* function, conferred by the *Kit^W-41J^* allele -have impaired endogenous mouse HSCs, allowing engraftment of human HSCs without irradiation -support better human erythropoiesis and platelet formation

## Improved Erythropoiesis and Megakaryopoiesis in Current Immunodeficient Mouse Models

Despite the successes seen over the last few decades in improving immunodeficient mouse models, significant challenges still remain. For example, NSG mice only allow low human myeloid cell development with high proportion of immature B cells (56, 57) with minimal production of antigen-specific IgG class antibodies (58) and very reduced production of erythrocytes (59). In fact, human cells that have proven most difficult to generate in these immunodeficient mice have been the erythrocytic and megakaryocytic lineages in which their *de novo* production from human hematopoietic stem and progenitor cells (HSPCs) normally peaks at 5–7 weeks following the xenotransplantation. This skewing in the lineage development of human cells could be multifactorial. The insufficient cross-reactivity of mouse cytokines to human cells may explain, at least in part, the poor development of these cell lineages. Certainly, humanized mice do not allow an efficient production of human megakaryocyte erythroid progenitors (MEPs). However, this deficiency is not corrected in *Rag2*^−/−^*Il2ry*^−/−^ mice expressing human thrombopoietin (TPO). Interestingly, this mouse strain generated increased levels of human cell engraftment, as well as higher multilineage differentiation of HSCs, with an increased ratio of myelomonocytic *versus* lymphoid cells (38). In addition, injection of plasmid DNA-encoding human erythropoietin (EPO) and IL-3 was found to modestly improve human red blood cell (RBC) reconstitution in NSG mice that were engrafted with CD34^+^ HSPCs (60). It is worth to note that phagocytosis of human RBCs and platelets by murine macrophages has been shown to play a larger role in the elimination of such human cells. This could explain the absence of these cell types in the peripheral blood of the humanized mice. Interestingly, human late-stage immature erythroid cells (CD235a^+^CD45^−^ nucleated normoblasts) are normally detected in the mouse BM; however, as these cells reach their maturity to form RBC, they are efficiently eliminated by murine macrophages (59, 61). As previously mentioned, NOD SIRP*α* is capable of cross-reacting with human CD47, suggesting that the rejection of human RBCs in humanized mice is unlikely to be induced by the inability of human CD47 to interact with the recipient SIRP*α*. Indeed, it was demonstrated that the rejection rates of human RBCs were significantly higher than CD47 knockout mouse RBCs, indicating that the rejection of human RBCs in immunodeficient mice is mainly induced by CD47-SIRP*α* independent mechanisms, such as xenoantigens present on human RBCs that can activate host macrophages (62, 63). Interestingly, intravenous injection of liposome-encapsulated clodronate into mice was shown to deplete recipient macrophages, resulting in robust, albeit transient, generation of both human RBCs and platelets (59, 61).

The recent generation of NSGW41 (with partial deficiency in Kit function) mice (**Table 1**) has definitely helped to improve human erythropoiesis and platelet formation in murine microenvironment (55, 64). Furthermore, human thrombopoiesis was observed in these mice following xenotransplantation, with increased proportions of human mature thrombocytes and erythroblasts in the murine BM. In addition, the morphology and composition of *de novo* generated human erythroblasts were found to be similar to that observed in the human BM. The most likely mechanism resulting in enhanced BM erythropoiesis in kit mutant mice is the cell intrinsic impairment of erythropoiesis in these mice (65). Interestingly, human erythro- and megakaryopoiesis were also found to be enhanced in an another recently developed immunodeficient mouse model (namely MISTRG mice), following the xenotransplantation of human HSPCs (66). The authors of this work postulated that the expression of human cytokines from the endogenous murine loci (replacing murine encoding cytokines), by providing a more physiologic expression of human cytokines, synergistically promotes a more competitive human hematopoiesis including the efficient development of human RBCs and platelets (66).

## Immune Innate Cell Differentiation Outputs Are Much Needed

In a healthy human adult individual, myeloid cells comprise ~55–70% of all leukocytes both in the peripheral blood and BM. As discussed earlier, the current common immunodeficient mouse models poorly recapitulate the normal myelopoiesis that is observed in human hematopoietic tissues, particularly the human macrophages that are poorly generated in mice. Cytokines play a key role not only in the maintenance of human HSCs but also in their differentiation to mature myeloid progeny. One of the main reasons responsible for poor human myelopoiesis in mice could be the evolutionary divergence between human and mouse cytokines, that are species-specific (*i.e.*, the mouse cytokines do not function in human cells). Early attempts were made to resolve these limitations which included the direct delivery of human cytokines (recombinant cytokines or plasmids encoding for cytokines) into the immunodeficient mice (60, 67, 68). However, these methods are expensive and not suitable for long-term experiments. Further quest for a “better” humanized mouse model led to the development of transgenic mice that are generated by overexpression of human cytokine-encoding genes under the control of a strong constitutive promoter. The NOD-SCID-SGM3 and NSG-SGM3 mouse strains (see **Table 1**) that overexpress SCF, GM-CSF, and IL-3 under the cytomegalovirus (CMV) promoter have been shown to increase the level of human myeloid cells upon engraftment with human HSPCs (43, 69). The latter mouse strain was also found to facilitate a more rapid reconstitution of T cells, with improved B cell differentiation and increased levels of NK cells as compared to the parental NSG strain. However, the supra-physiological levels of these cytokines in these mice were shown to almost abolish the human platelets that are normally detected in NSG mice at a low level, and more importantly cause exhaustion of the engrafted human HSCs (69). A closely related strain such as NOG-*IL3*/*CSF2* (NOG-EXL) mice that overexpresses human IL-3 and GM-CSF, was also shown to have increased levels of human myeloid cells, in particular basophils, mast cells, and myeloid derived dendritic cells (DCs). However, this mouse strain appears to preserve the human HSPCs (44). It is unclear why this mouse strain does not seem to exhaust the human HSPCs. One of the reasons for such a behavior could be the level of the transgene expression that is driven by different promoters in these models (*i.e.* SR*α* instead of the CMV promoter) (44). However, BRG mice with human *IL3* and *CSF2* knock-in genes (BRG-*IL3*/*CSF2*) were found to have no significant increase in human T, B, and NK cells as well as human HSPCs, CD34^+^CD33^+^ myeloid progenitors, total CD33^+^ myeloid cells, CD14^+^ monocytes/macrophages and CD66^+^ granulocytes in the bone marrow, blood, and spleen of these mice (36). However, BRG-*IL3*/*CSF2* mice were shown to support the development and replacement of human alveolar marcophages (36). This may suggest that additional factors other than only the lack of cross-reactivity of the cytokines between the species, are also important for generating robust human myelopoiesis in hematopoietic tissues in mice. In another model, a transgenic NOG mouse was generated using human *IL6* cDNA under the control of the CMV promoter (NOG-*IL6*). These mice generated significant levels of CD14^+^HLA-DR^lo/neg^ human monocytic myeloid derived suppressor cells, following HSPC xenotransplantation. In addition, infiltration of human CD163^+^ tumor-associated macrophages was evidently detected in a head and neck squamous cell carcinoma engrafted model (45).To specifically increase macrophage development, human macrophage colony stimulating factor (M-CSF; NSG-*CSF1*) gene was overexpressed in NSG mice (50), which when crossed with NSG-SGM3 mice, led to the generation of NSG-SGM3-*CSF1* (QUAD) mice. Interestingly, these mice showed increased human myeloid and mature macrophage development when engrafted with human HSPCs (33). In order to further refine the expression of these critical cytokines, Rongvaux and colleagues used 129×BALB/c-*Rag2*^−/−^*Il2r*γ^−/−^ mice to knock-in human *IL3*, *CSF2*, *TPO* (MITRG) and in addition *SIRPA* (MISTRG mice; see **Table 1**) in these mice. These mice allowed a robust development of human myelopoiesis and showed a strong human innate immune response to viral and bacterial infections. The authors also observed a functional human NK cell development that could be attributed to the production of IL-15 by the human monocytes developed in these mice (41). However, these mice eventually developed severe anemia as a result of the high human cell engraftment, which resulted in shorter lifespan of these mice. This was due to the destruction of mouse RBCs as a result of the enhanced human myeloid cell function (41).

Another innate immune cell population that displays suboptimal development in immunodeficient mice are the NK cells. Early effort to boost NK cell formation was demonstrated when BRG mice engrafted with human HSPCs were injected with recombinant IL-15/IL-15-receptor complexes that induced extensive NK cell proliferation and differentiation. This resulted in accumulation of CD16^+^KIR^+^ NK cells in these mice (70). This study led to the generation of NOG-*IL15* mice (see **Table 1**). The high levels of circulating IL-15 cytokines in these mice were shown to support the survival of human peripheral blood-derived NK cells (46). Interestingly, human *SIRPA* and *IL15* knock-in RAG (SRG-*IL15*) mice have more physiological levels of IL-15 but upon poly I:C stimulation these mice can express high levels of IL-15, resulting in an efficient functional human NK cells that can mediate antibody-dependent cellular cytotoxicity (40). Furthermore, compared to the all above innate immune cells, human CD123^+^ plasmacytoid and myeloid CD11c^+^ DCs are efficiently developed in most immunodeficient mouse models (71) even in NOD-*Scid* mice (72) without the requirement of exogenous human cytokines or the use of additional human transgene expression. Nevertheless, administration of recombinant FLT3-L was demonstrated to induce HSPC differentiation towards fully functional human CD141^+^ and CD1c^+^ DCs (73). As CD141^+^ DCs are endowed with the capacity to cross-present viral antigens after their uptake of necrotic virus-infected cells, produce high levels of IL-12, and induce Th1 responses, CD1c^+^ DCs, on the other hand, play a key role in driving adaptive immune responses to extracellular pathogens, owing to their capacity to promote Th2 and Th17 responses. Both DC fractions can serve important bridges for the elaboration of more complex human immune response studies in these immunodeficient mice.

## Can Human B Cells Fully Develop in Mice?

Recent efforts to further enhance human immune system development and function in humanized mouse models have focused on improving specific innate immune cell lineages. An early study using mice with partial immunodeficiency has shown incomplete human B cell development, with large proportion of engrafted B cells having an immature (CD10+) and B1-type phenotype (CD5+) (74). Subsequent reports that used NSG mice demonstrated improved B cell function including class switching in which the VH‐DH‐JH composition was seen to be similar to the mice engrafted with cord blood cells. In these mice, the production of IgM, IgA, and IgG antibodies were also observed (75, 76). The general view in the field appears to be that B cell maturation in human HSPC engrafted mice increases with time, reaching up to 60% of the B cells having a mature phenotype (CD20^+^CD10^−^) after 24 weeks (57). Human *de novo* B cells isolated from NSG mice have also been shown to undergo somatic hypermutation (77). In fact, several studies have observed the production of antigen-specific human IgM and IgG antibodies in humanized mice in response to infection or immunization with varied degrees of responses (74, 78–80). Some of these humanized mice generated functional neutralizing antibodies in a model of Dengue virus infection (79). Furthermore, these mice have also been used to test the selection process of human autoreactive B cells. Indeed, Lang and colleagues used BRG mice that express a synthetic self-antigen specific constant region of human Ig*κ* and demonstrated that developing human autoreactive (*κ*+) B cells undergo central tolerance by both receptor editing as well as clonal deletion (81). Other studies have supported this notion of autoreactive human B cell development, therefore confirming the intact central B cell tolerance in immunodeficient mice (82, 83). Therefore, humanized mouse models could also be used to elucidate some aspects of the molecular mechanisms of autoimmunity.

To further enhance class switching and somatic hypermutation, Flavell and colleagues generated immunodeficient *Rag2^−/−^Il2rγ^−/−^* mice in which *IL6* was integrated into its orthologous mouse locus (39). These RGSKI-*IL6* mice expressing human IL-6 not only allowed the improvement of thymopoiesis and peripheral T cell reconstitution but also showed significant increase in total IgG and antigen-specific IgG levels. The authors of this work also observed higher frequencies of memory and IgG producing B cells. Interestingly, once these mice were challenged with ovalbumin (OVA), the OVA-specific antibodies displayed high frequency of somatic mutations (39). This mouse model then led to the development of the MISTRG6 mice, that enabled further improvement of B cell development. Consequently, MISTRG6 mouse model was used to investigate the entire spectrum of human plasma cell neoplasia (42). Recently, IL-6 transgenic strain was created on the NSG background using human *IL6* BAC (NSG-IL6) (33). These NSG-*IL6* mice engrafted with human HSPCs displayed increased levels of CD3^+^ and Th17 T cells compared to NSG mice. In addition, higher plasma levels of IgM and IgG were observed in these mice (33). In summary, human IL6-expressing immunodeficient mice could be a useful tool to evaluate the elicitation of antigen-specific antibody responses.

## Human Thymopoiesis: A Proper T Selection With Human Leukocyte Antigen-Restricted Immune Responses in Mice?

Human conventional T cells’ response relies on the ability of their TCRs to recognize a specific peptide (and subsequently bind) in the context of an MHC molecule that is solely expressed in the thymus during their initial developmental phase. To examine human immune responses in immunodeficient mice, leukocytes can be engrafted by intravenous or intraperitoneal injection of human peripheral blood mononuclear cells (PBMCs) into mice. This model is less laborious and time consuming compared to the *de novo* generation of leukocytes through BM xenotransplantation using HSPCs. Although human leukocytes, in particular T cells, can be found circulating in the murine peripheral blood within days following the injection; however, this process can lead to a rapid xenogeneic graft *versus* host disease (GvHD) (84, 85). One of the major drawbacks of this system includes the limited T cell repertoire and prevalence of uncontrolled B cell activation with the production of human xenoreactive antibodies (86, 87). However, some of these concerns can be circumvented by using new mouse strains that are for the major histocompatibility complex (MHC) class I/II [such as, NSG-*B2M^null^* and NSG*-*(*K^b^D^b^*)*^null^*(*IA^null^*) mice]. Injection of human PBMCs into these strains enables long-term engraftment of human CD4^+^ and CD8^+^ T cells without causing acute GvHD (49, 51). Alternatively, xenogeneic GvHD in immunodeficient mice can be eliminated by a more classical approach that enables the reconstitution of human immune system including T cells through the transplantation of HSPCs. This method enables the generation of human T cells that can be detected in murine thymus at around 12 weeks, depending upon the level of human cell engraftment in the mouse BM. However, this process can be accelerated when human HSPC transplantation is performed in new-born mouse (88–90), that perhaps occurs as a result of a faster seeding of primitive precursors in the mouse thymus. On the other hand, adult NSG-SGM3 mice also have improved T cell development (69). The exact mechanism and the particular changes responsible for this improved lymphopoiesis remain unknown. It is worth to note that this process still requires an extended developmental time, but interestingly different human T cell populations and diverse T cell receptor variable beta chain (TCR V*β*) repertoire can be generated in these mice (89, 91, 92). However, one of the caveats in human T cell development in humanized NSG murine microenvironment is that Th1, Th2, and Th17 cells were found at very low levels (51, 93), although the quantification of these cells was performed following an *in vitro* stimulation.

Development of human T cells in NOD-*Scid* and Rag-deficient derived mice undergoes maturation as well as selection *via* recognition of mouse MHC molecules expressed by thymus mouse epithelial cells and DCs. As these human T cells are educated and restricted solely to murine MHC, therefore they are not suitable for studies investigating T cell responses with human antigen presenting cells (APCs), which express human MHC receptors. To acquire the immune sensitivities with high diversity for human cells, education of T cells must be performed by the human MHC molecules in a murine environment. This major issue was addressed by transplanting human HSPCs isolated from human fetal liver into NOD-*Scid* mice that were implanted with human liver and thymus (under the mouse kidney capsule) from the same fetus (BLT model). This BLT model demonstrated that human T cells developed in the human thymus (implanted in mouse) were human Human Leukocyte antigen and generated immune response, with development of a mucosal system similar to humans (94–96). Despite these advantages, this model is still time consuming and technically difficult, and mice can still develop xenogeneic GvHD (97). In a recent development, NSG-*HLA-A2/HHD* mice expressing human HLA class I showed the development of virus-specific HLA-A2-restricted human T cell responses to Dengue virus infection (79). In a subsequent study, this mouse model generated functionally HLA-restricted cytotoxic T cells against Epstein–Barr virus (EBV) virus, therefore suggesting that the recognition and education of human HLA can take place in NSG mouse with a functional thymus. Furthermore, interferon gamma (IFN*γ*)-producing human T cells against multiple EBV HLA-A2 epitopes were observed showing similar patterns of reactivity to that detected in human infections, including the development of CD8^+^CD45RO^+^ memory T cells (51). These two reports described that HLA expressed in murine thymus allows human T cells to be positively selected and in fact these cells can induce HLA-restricted immune responses in murine environment. There are other HLA-transgenic immunodeficient mice that have recently been established, such as NSG mice expressing HLA-A*0201 and A*2402. This study showed that human cytotoxic T cells upon stimulation with Wilms’ tumor 1 (WT1) peptide in *in vitro* conditions, produced IFN*γ* and cytotoxic activity against leukemic cells in an antigen-specific and HLA-restricted manner (52). In another report, NSG‐*HLA‐A2/DR1* mice were used to study human antiviral adaptive responses during hepatotropic virus infection (98). It was reported that these mice allowed the engraftment of functional human Th1, Th2, and Th17 T cells, and some of these T cells were polyfunctional effector cells (CD62L^−^ CCR7^-^HLA‐DR^+^). Interestingly, these cells secreted a variety of cytokines such as IFN*γ*, tumor necrosis factor alpha (TNFα), IL-4 and IL-17 (98). The HLA‐*DR4* single knock‐in mice (DRAG mice; NRG-*HLA-A2-DR4*; see **Table 1**) offer further improved polyfunctionality and antigen specificity of effector CD8^+^ cells over the HLA‐A2 NSG mice (99). Interestingly, these mice were also shown to allow the generation of high levels of CD4^+^CXCR5^+^PD1^+^ T follicular helper cells that were efficiently disseminated into the gut‐associated lymphoid tissues (100). Therefore, HLA class I and II transgenic NSG mouse models may serve as a preclinical tool to develop effective immunotherapy against human malignancies.

## Is More Humanization of Immunodeficient Models Needed?

Even though the above mentioned immunodeficient mouse models have been useful, they still present various challenges. One of the biggest caveats is cross-species differences that exist between the engrafted human cells and mouse microenvironment (including immune-microenvironment) they reside in, and how this may influence various environmental clues that mediate cell–cell interactions, hematopoietic cell homing, survival, and expansion, among others. Recently, integration of biomaterials with immunodeficient mouse models has enabled researchers to generate a more physiological human microenvironment namely ‘humanized ectopic scaffold niche’, that provides architectural support for cell attachment and subsequent human BM-like tissue development. These humanized scaffold-based approaches can potentially enable the researchers to closely mimic the multicellular aspects of the human bone in a mouse model (101–104). This approach can provide an important tool to potentially generate patient-specific human microenvironment including endothelium in immunodeficient mice that can be used to unravel not only the role of human tumor microenvironments, disease pathology but can potentially be used to gather physiological response to various immunotherapies.

## Profiling of Cancer Immunotherapies in Mouse Models With Human Immuno-Microenvironment

**Figure 2:**
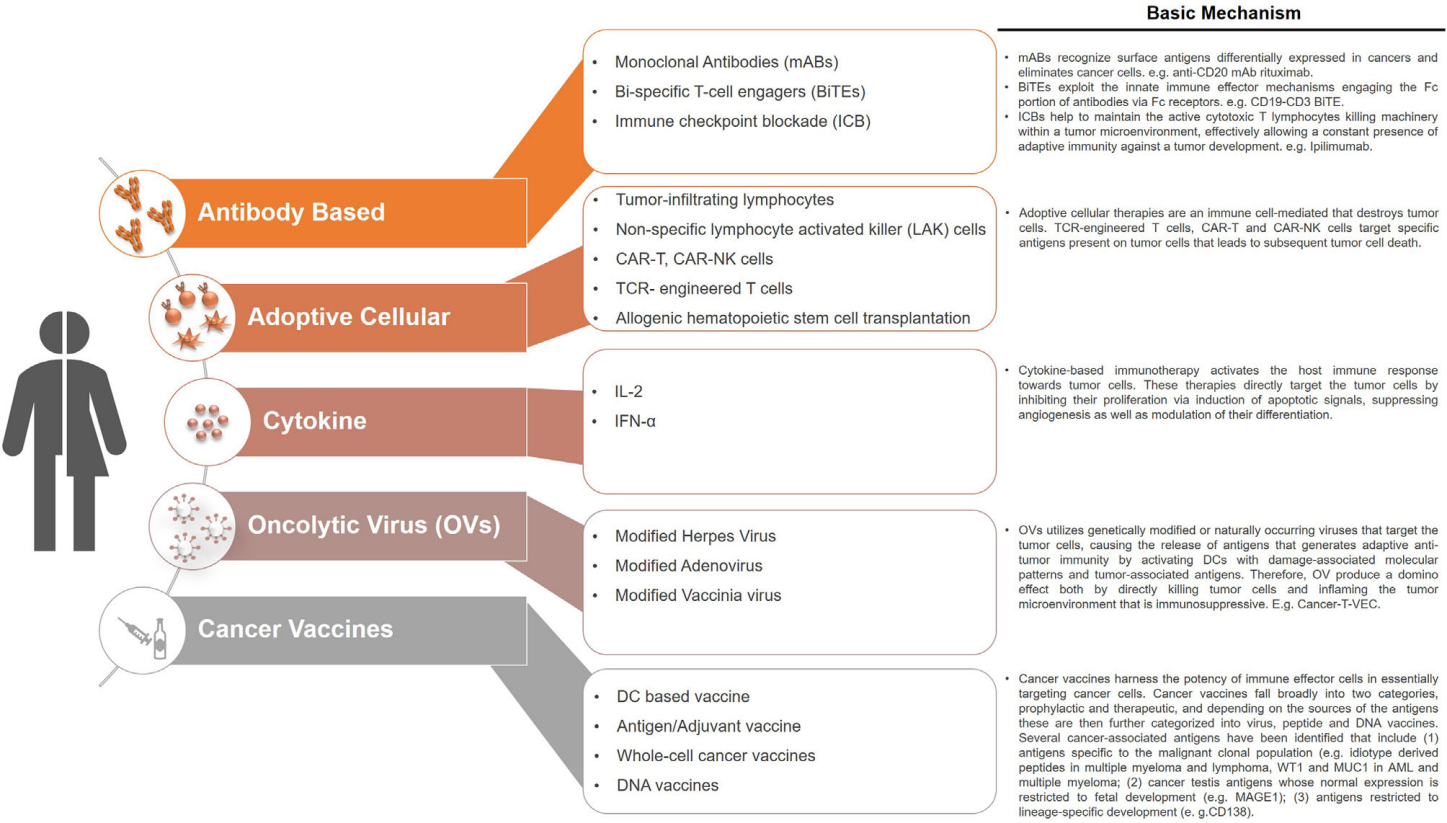
Current immunotherapeutic strategies in cancers. Cancer immunotherapies target the patients own immune system by restoring and stimulating various immune components that ultimately had the potential to inhibit cancer growth and/or eradicate cancer. The various types of immunotherapies that are currently being used in the clinic or are under development include antibody-based immunotherapy, oncolytic virus therapy, cytokine therapy, cellular therapy, cancer vaccines. These therapies are also being used as combination therapies that target multiple immune components or with other established therapeutic options such as chemotherapy.

Immunotherapies are considered to be the cornerstone of current therapeutic strategies against cancer, along with surgery, radiation, cytotoxic chemotherapy, and targeted drug therapy (105). Current strategies of immunotherapies are summarized in **Figure 2**. Intrinsic therapeutic modulation of the adaptive and innate components of the human immune system to enhance recognition that subsequently leads to improved response against tumors is widely adopted strategy in the clinical fight targeting different types of cancers. There are more than 3,000 types of cancer immunotherapies either in development or currently assessed in clinical trials, that include antibody-based immunotherapy, oncolytic virus therapy, cytokine therapy, cellular therapy (CAR-T, CAR-NK, TCR-T and DC-based), cancer vaccines and the combination of immunotherapy that targets multiple immune components (106). Although, these therapies focused on unleashing the immune system are considered a breakthrough; however, durable clinical responses only occur in a subset of patients. Furthermore, the development of novel therapeutics is tightly restricted by the use of human samples before moving to clinical trials, which is generally slow and costly. Therefore, considerable scientific resources are still needed in preclinical research to identify novel and improved approaches to cancer cell‐specific immune response as well as testing of therapeutic strategies. Over the last half century, syngeneic mouse tumor models were the major *in vivo* systems used for understanding cancer immunology and associated immunotherapies. However, the paradigm shifted with the dramatic advances in our understanding of the interactions between tumor development and the immune microenvironment. Furthermore, immunotherapies such as immunomodulatory agents do not always react with mouse molecules due to cross species barriers. Together all these factors led to the increased humanization of immunodeficient mouse models in which both the human immune cells and human tumors are cohabiting with each other. However, these models are quite complex, and the fact that obtaining human immune cells as well as the malignant cells from the same patient still remains difficult, researchers are currently using HLA mismatched cord blood derived HSPCs or healthy donor derived PBMCs for humanization along with patient derived tumor cells or cancer cell lines (**Figure 1**). Even with these caveats, the use of these humanized models has emerged as an invaluable tool in providing a system not only to study normal human immune system interactions with the malignant cellular components but have also accelerated the development of the cancer immunotherapies. These preclinical model systems have enabled to better predict the clinical outcomes of immunotherapy regimens before moving to clinical trials.

## First Generation Humanized Mouse Models

To date, one of the most popular immunodeficient mouse models used in the field of cancer biology as well as immuno-oncology is the NSG mice. These models have enabled the generation of patient derived xenografts (PDXs) particularly in hematological malignancies where they have played an instrumental role in the identification and understanding of leukemia initiating cells (101, 107) (**Figure 1**). Furthermore, these humanized mouse models have enabled personalized tests on the sensitivity and specificity of the tumors to many therapeutic regimens. Among various cellular immunotherapies, anti-CD19 directed CAR-T cells that were shown to eliminate the CD19^+^ B cell leukemia in NSG PDX model, resulted in prolonged survival of these mice, and this data set the basis for the first clinical trial and later on, approval of the first CAR T cell products by the FDA (108). In a recent development focusing on cellular therapies that could pave the way for off-the shelf cellular therapies, Li and colleagues used NSG mice to demonstrate the feasibility of using the next generation iPSC-derived CAR-NK cells as a treatment option for mesothelin-over expressing ovarian tumors (109). Monoclonal antibody (92R) tested in NSG mice to target T cell acute lymphoblastic leukemia cells strongly inhibited tumor growth *via* binding to the C-C chemokine receptor type 9 (CCR9) N-terminal domain. This leukemia killing response was observed in a model with compromised NK and complement activities, suggesting that other mechanisms (such as phagocytosis or apoptosis) might also be playing a role in tumor destruction (110).

One of the major drawbacks of these PDXs established in NSG model is the lack of immunosurveillance by the host. Considerable efforts that led to the generation of NSG-PBMC models have been used widely to study single and combination therapies. For example, IgG-based BCMA-T cell bispecific antibody (EM801) targeting B cell maturation antigen (BCMA) induced myeloma cell death by autologous T cells (111). Anti-tumor activity of ICBs (nivolumab, pembrolizumab, atezolizumab, cetuximab, and urelumab) was also observed in the PBMC-humanized mouse models (112–114). Interestingly, the differences in tumor growth were not significantly different between mono- and combination therapies; however, the humanized model was suitable to develop mechanistic studies that supported this combination in the subsequent clinical studies (113). This initial experience represented the first proof of concept report to demonstrate that immunotherapy combination strategies can be modeled in preclinical settings involving humanized mouse models. On the other hand, multiple DC-based vaccine tested in NSG-PBMC mouse model enabled assessment of melanoma antigen recognized by T cells-1 (MART-1)-specific immune responses and suppressive functions on melanoma cells (115). Although the ease of generating these mice allows for their use in tumor-bearing PDXs, challenges arise with regard to the onset of xenogeneic robust GvHD, which occurs concomitant with T cell expansion (116, 117). The limited window of time (approximately 2–3 weeks) for taking observation could enable the investigations into T cell-mediated anti-tumor mechanisms; however, the use of NSG or NOG MHC knockout mice or alternatively xenotransplantation of purified CD8^+^ T cells may enable long-term immune response studies (49, 118, 119). Using ICB-based (PD-1) immunotherapy in these mouse models, specific CD8^+^ T cell population was identified that produced anti-tumor effect in an HLA restricted manner without needing to worry about xenogeneic GvHD (120).

HSPC-engrafted human immune system (HIS) models provide powerful tools for long-term studies and are increasingly being used for understanding T cell-mediated immunotherapy mechanisms. The caveat of this HSPC-HIS (NSG and NOG) model is the biased differentiation of HSPCs towards the lymphoid lineage, with the limited development of myeloid cells. However, despite this limitation, HSPC-HIS mice have successfully provided a model system to study T cell-dependent immunotherapy mechanisms (such as blocking PD-1, CTLA-4 alone or in combination with oncolytic viruses) and benefit from long experimental time scales (121–123). In cord blood derived HSPC-generated humanized mouse model, PD-1 blocking (using nivolumab) inhibited the growth of MDAMB-231 tumor cells and CRC172 tumor cells by enhancing anti-tumor T cell response, increasing GrB^+^ or IFN*γ*^+^ CD8^+^ cells in tumors and reducing frequency of Treg and myeloid cells. However, combination of histone deacetylase (HDAC) inhibitors OKI-179 and nivolumab further inhibited cancer cell growth, therefore indicating that HDAC inhibitors could improve anti-tumor immune responses in cancers (124). In another study, a combination of anti-CD278 mAb and cyclophosphamide controlled the growth of breast cancer in NSG humanized mice. Administration of a neutralizing anti-CD278 mAb reduced human Treg proportions and numbers and improved CD4^+^ T cell proliferation therefore highlighting the crucial implication of innate immunity in treatment efficacy, opening new perspectives for the treatment of breast cancer (125). In another study, transplantation of allogeneic but HLA partially matched donor HSPCs and tumor cells (non‐small cell lung cancer, sarcoma, bladder cancer, and triple‐negative breast cancer) in NSG mice demonstrated significant tumor growth delay following pembrolizumab (anti‐PD‐1) therapy (121). In a different approach, Haworth and colleagues reconstituted NSG mice with human HSPCs that lead to the generation of matured human CD3^+^ T cells, which can be isolated, genetically modified and then reinfused into the same mice (89). It is worth to note that no clinical symptoms of GvHD were observed in these mice, and therefore this model might represent a better system for long term evaluation of cellular immunotherapies such as CAR-T cells.

## Second Generation Humanized Mouse Models

Until recently, the development of more balanced humanization by improving the differentiation and survival of various human immune cell populations has been a major goal; the function of these human immune cells in murine system was overlooked. These issues have led to the development of so called “second generation” immunodeficient models that express human transgenes. These models (*e.g.* NSG-SGM3, NOG-EXL, NSG HLA-A2 and MISTRG) not only allow more myeloid and NK cell development apart from lymphoid reconstitution, but also enable the function of engrafted human immune cells thus, improving the utility and translatability of these models in immunotherapy related studies (**Figure 1**). NOG-IL2 transgenic mice were able to overcome the previous problems and recapitulated the clinical responses of patient-derived TILs (126). In a preclinical model using HSPC transplanted humanized BRGS mice, Capasso and colleagues established breast cancer and colon cancer PDXs, and showed strong suppressive function of PD-1 blockade that resulted in tumor growth inhibition with increased CD8 IFN*γ*^+^ tumor infiltrating T cells (124). Transgenic mice that express the human HLA-A2 allele, commonly detected in the Caucasian population, were developed to study immunologic interactions between human T cells and tumor cells (51, 127). Studies investigating the antigen-specific HLA-restricted response against Epstein–Barr virus (EBV)-associated tumors, found CD8^+^ (and CD4^+^) effector-to-target ratio-dependent cytotoxicity against EBV-infected lymphomas (128). The humanized transgenic NSG-SGM3 mice have shown enhanced human Treg cell differentiation, and these Treg cells were functional and were able to suppress the proliferation of T cells in PDX generated from HSPCs (93). Notably, similar data of T cell suppression by Tregs was reported in PDX models for autoimmune conditions such as aplastic anemia (129).

MISTRG mice that have recently been developed show robust differentiation of human innate as well as adaptive immune cells and have been used to study acute myeloid leukemia and diffuse large B-cell lymphoma (41, 130). Interestingly, human macrophages generated in MISTRG infiltrated a human tumor xenograft in a manner resembling that observed in tumors obtained from patients (41). Therefore, these MISTRG mice recapitulate the role of macrophages in tumor development and fulfil a critical need for models that enable study of the interaction between human tumors and human innate immune microenvironment. The major advantage of these humanized systems is the ability to observe the immune surveillance against human cancer tissues. However, one caveat of these models is that the reconstituted human immune system lacks physiological maturity due to the absence of human thymus tissue. To address these concerns, BLT mouse model developed, harbors an almost intact functional human immune system therefore providing a powerful tool to study cancer immunotherapy (131). In a recent study, implantation of human lung tissue into NSG mice pre-humanized with BM/liver/thymus resulted in robust antigen-specific humoral and T cell responses against cytomegalovirus infection (132). In a different study, Jin and colleagues developed an autologous NSG BLT-based model with a fully competent human immune system. Following the humanization, mice were transplanted with edited fetal HSPCs that recapitulated human B-ALL like disease. This model system was used to test autologous anti-CD19-CAR T cells. This preclinical model, though complex in its generation, was highly adapted to evaluate human CAR T cell efficacy, resistance and toxicity (133). These second-generation humanization strategies provide a promising tool for understanding the tumor immune microenvironment interactions and enable greater translational potential and expanded opportunities for therapeutic evaluation. However, BLT mice do require human fetal tissues which are difficult to source especially if needed in large quantities. However, surplus neonatal tissue can be a good viable alternative that can be used for mouse humanization (134).

## Future Developments

Although immunodeficient mouse models along with increased humanization have been instrumental in gaining understanding of the cancer biology and develop various targeted immunotherapies, these models do have limitations that need to be addressed to create more appropriate model system that will fulfil the need of current and future research. There is an urgent need to develop new strategies that will enable us to further the humanization of these mice, particularly focusing on the development of comprehensive and functional human immune system. One of the ways to improve on the current sub-optimal development of human immune cells is to further characterize and identify the cytokines as well as growth factors that do not cross react between mouse and human. It is also important to identify and integrate novel approaches that would enable autologous experiments, where malignant tissues and immune cells from the same individual are used, that would provide more accurate understanding of disease progression, tumor-immune interactions and efficacy of personalized therapeutic options. Improving the humanization of these mice will also enable us to shed more light on the basic tumor immunology, particularly the roles of the immunosuppressive tumor immune-microenvironment and tumor neoantigens that often shape the development of cancer and influence therapeutic efficacy. Altogether, having an appropriate humanized preclinical model will enable speedier assessment of the efficacy and safety of novel therapeutic approaches and will ultimately provide a faster bench to beside transition.

## Author Contributions

All authors listed have made a substantial, direct, and intellectual contribution to the work and approved it for publication.

## Funding

This work was supported by the Francis Crick Institute (to DB), which receives its core funding from Cancer Research UK (FC0010045), the UK Medical Research Council (FC0010045), and the Wellcome Trust (FC001045) and by the UK Biotechnology and Biological Sciences Research Council (BB/S017097/1; to FA-A). We would like to thank Blood Cancer UK for their support to SAM.

## Conflict of Interest

The authors declare that the research was conducted in the absence of any commercial or financial relationships that could be construed as a potential conflict of interest.

## Glossary

**Table:**

*Prkdc*	gene encoding for protein kinase DNA-activated catalytic subunit which is required for the non-homologous end joining pathway of DNA repair during V(D)J recombination. Mutation of *Prkdc* leads to V(D)J recombination defects.
*Sirpa*	gene encoding for signal regulatory protein alpha; SIRPα acts as inhibitory receptor and interacts with the transmembrane protein CD47 also called the “don’t eat me” signal. This interaction negatively controls effector function of innate immune cells such as host cell phagocytosis.
*Rag1* or *Rag2*	recombination activating gene 1 or 2; the protein encoded by these genes are involved in antibody and T-cell receptor V(D)J recombination.
SCF	stem cell factor, is a cytokine that binds to the c-KIT receptor, and it plays a role in the regulation of HSCs in the niche by increasing the survival and self-renewal of HSCs.
GM-CSF	granulocyte macrophage colony stimulating factor; it stimulates stem cells to produce granulocytes and monocytes.
SR*α*	Simian virus 40 early promoter and the R segment and part of the U5 sequence of the long terminal repeat of human T cell leukemia virus type 1
*CSF2*	gene encoding for GM-CSF
FLT3-L	fms-related tyrosine kinase 3 ligand; is a hematopoietic cytokine; it stimulates the survival, proliferation, and differentiation of various blood cell progenitors.
CAR-T, CAR-NK	chimeric antigen receptor T*-*cell, chimeric antigen receptor NK-cell; CARs are receptor proteins that have been engineered to give T or NK cells the new ability to target a specific protein. The receptors are chimeric because they combine both antigen-binding and T/NK-cell activating functions into a single receptor.
